# Comparison of the relative muscle volume of triceps surae among sprinters, runners, and untrained participants

**DOI:** 10.14814/phy2.14588

**Published:** 2020-10-10

**Authors:** Atsuki Fukutani, Yume Tsuruhara, Yuto Miyake, Kenji Takao, Hiromasa Ueno, Mitsuo Otsuka, Tadashi Suga, Masafumi Terada, Akinori Nagano, Tadao Isaka

**Affiliations:** ^1^ Faculty of Sport and Health Science Ritsumeikan University Kusatsu Shiga Japan; ^2^ College of Sport and Health Science Ritsumeikan University Kusatsu Shiga Japan; ^3^ Graduate School of Sport and Health Science Ritsumeikan University Kusatsu Shiga Japan; ^4^ Graduate School of Health and Sport Science Nippon Sport Science University Setagaya Tokyo Japan; ^5^ Research Fellowship for Young Scientists Japan Society for the Promotion of Science Chiyoda Tokyo Japan

**Keywords:** lateral gastrocnemius, magnetic resonance imaging, medial gastrocnemius, plantar flexors, soleus

## Abstract

Muscle hypertrophy is considered more prominent in fast‐twitch than in slow‐twitch muscles. This leads to the hypothesis that the relative muscle volume of the medial gastrocnemius (MG) and lateral gastrocnemius (LG) becomes larger than that of the soleus (SOL) in highly trained participants because MG and LG include more fast‐twitch muscles than SOL. Thus, we compared relative muscle volume among highly trained sprinters, long‐distance runners, and untrained participants to examine whether the above hypothesis is correct. Magnetic resonance imaging was used to calculate the muscle volume of MG, LG, and SOL from 126 participants. The total muscle volume of the three muscles and the relative muscle volume of each muscle with respect to the total muscle volume were calculated. The total muscle volume was significantly larger in the sprinters than in the long‐distance runners and untrained participants. The relative muscle volume of MG was significantly larger in the sprinters than in the long‐distance runners and untrained participants and that of SOL was significantly smaller in the sprinters than in the long‐distance runners and untrained participants. These results indicate that the relative muscle volume can vary among participants, possibly due to fiber type‐dependent muscle hypertrophy.

## Graphical abstract

The relative muscle volume among groups was different. This result indicates that muscle size (volume/physiological cross sectional area) should be measured individually to calculate the individual muscle force from the joint torque based on the relative muscle size among synergistic muscles.

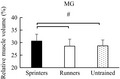

## 1 INTRODUCTION

Muscle architecture is important information because muscle function is closely related to muscle architecture. For example, muscle force or joint torque is mainly determined by muscle cross‐sectional area (CSA) or muscle volume (Fukunaga et al., 2001; Ikai & Fukunaga, 1968). In addition, shortening velocity of the muscle is a function of muscle length (Bodine et al., 1982). Therefore, it is crucial to accurately evaluate information regarding muscle architecture.

The human triceps surae is mainly composed of three muscles: medial gastrocnemius (MG), lateral gastrocnemius (LG), and soleus (SOL). Plantar flexion torque is mainly produced by the collaboration of these three muscles (Fukunaga et al., 1992). In other word, it is difficult to distinguish the contribution of individual muscles on plantar flexion torque based on joint torque measurements. Due to this limitation, we usually estimate the contribution of each muscle from its relative muscle size. In concrete, if the relative muscle size of MG is 30% with respect to the total muscle size (i.e., the sum of MG, LG, and SOL), the joint torque produced by MG is considered 30% with respect to the measured plantar flexion torque. This estimation is based on the concept that the magnitude of joint torque (or muscle force) is determined by the muscle volume (or CSA) (Fukunaga et al., 1992, 2001; Ikai & Fukunaga, 1968). This estimation method makes it possible to examine the tendon stiffness and/or force–length–velocity relationship of individual muscles, although the force produced by each muscle cannot be measured directly and noninvasively in humans (Burgess, Pearson, & Onambélé, 2009; Kubo et al., 2000; Lichtwark & Wilson, 2007, 2008; Maganaris, Baltzopoulos, & Sargeant, 1998). When the above‐mentioned estimation method is to be applied, information on the relative muscle volume (or relative muscle CSA) with respect to the total muscle volume (or total muscle CSA) is needed. However, magnetic resonance imaging (MRI) is necessary to obtain these values, which is difficult to apply even in laboratory experiments. Thus, many studies have referred to the previous experiment which experimentally observed muscle volume and CSA using MRI (Fukunaga et al., 1992) to examine the behavior of each muscle separately (Burgess et al., 2009; Kubo et al., 2000; Lichtwark & Wilson, 2007, 2008). However, this estimation method makes sense only when the relative muscle volume is constant among participants. Specifically, if a participant has a relatively larger MG with respect to SOL, the estimated muscle force produced by MG derived from the previously reported ratio (Fukunaga et al., 1992) should be underestimated.

Regarding this point, it is generally considered that muscle hypertrophy is more prominent in fast‐twitch muscles than in slow‐twitch muscles (Oishi et al., 2002; Shi et al., 2007). Because SOL is mainly composed of slow‐twitch muscles, while MG and LG include slow‐ and fast‐twitch muscles equally (Johnson, Polgar, Weightman, & Appleton, 1973), one can speculate that highly trained participants have relatively larger muscle volume in MG and LG than in SOL. Therefore, the ratio of relative muscle volume might be different among participants. If this hypothesis is correct, the values obtained using the estimation method (i.e., applying the constant ratio to various participants) might be overestimated or underestimated.

Therefore, we measured the muscle volume from highly trained sprinters as the representative of highly trained (hypertrophied) participants, long‐distance runners and untrained participants as the representatives of nonhypertrophied participants to examine whether training status affects relative muscle volume. We hypothesized that highly trained sprinters have larger triceps surae than the long‐distance runners or untrained participants. This larger total muscle volume may be caused by selective muscle hypertrophy of fast‐twitch muscles (MG and LG) in sprinters, leading to the larger relative muscle volumes of MG and LG with respect to SOL, than in runners or untrained participants.

## 2 MATERIALS AND METHODS

### 2.1 Participants

In this study, 48 sprinters (age: 20.9 ± 1.9 years, height: 1.76 ± 0.05 m, body mass: 66.3 ± 5.0 kg, 100 m personal best time: 11.1 ± 0.4 s), 40 long‐distance runners (age: 19.8 ± 1.1 years, height: 1.71 ± 0.07 m, body mass: 55.7 ± 5.1 kg, 5,000 m personal best time: 902.6 ± 26.5 s), and 38 untrained participants (age: 21.6 ± 1.4 years, height: 1.71 ± 0.05 m, body mass: 65.5 ± 8.5 kg) were recruited (total number of participants = 126). The sprinters and long‐distance runners were involved in regular specialized training programs, while the untrained participants were not. The purpose and risks of the study were explained to each participant, all of whom provided written informed consent. The Ethics Committee on Human Research of Ritsumeikan University approved the study (BKC‐IRB‐2016‐047). This study was conducted according to the code of ethics outlined in the Declaration of Helsinki.

### 2.2 Protocols and measurements

All measurements were conducted on the right lower extremity of each participant. MRI (1.5T, SignaHDxt, GE Healthcare, Buckinghamshire, UK) was used to obtain images of muscle CSA of the triceps surae from all participants. The scanning protocol of MRI was as follows: fast spin echo, matrix 512 × 256, repetition time 600 ms, echo time 7.6 ms, slice thickness 10 mm, gap 0 mm, field of view 480 mm with a standard body coil. To obtain images of the triceps surae, the participants lay supine on the MRI bed with their hip and knee joints fully extended (0°) and their ankle joint angles fixed at 0**°** (anatomical position). Scan planes for the triceps surae muscles were perpendicular to the tibia.

### 2.3 Analyses

Obtained images were manually digitized by using an image analysis software (OsiriX Version 5.6, Osirix Foundation, Geneva, Switzerland). The muscle volume of MG, LG, and SOL was calculated by integrating the muscle CSAs attained from each image from the proximal to distal ends of each muscle. Adipose and connective tissue incursions were excluded as much as possible from each image of the CSA. Total muscle volume was calculated as the sum of the muscle volumes of MG, LG, and SOL. The relative muscle volume of MG, LG, and SOL was calculated as the relative value with respect to the aforementioned total muscle volume.

### 2.4 Statistics

One‐way analysis of variance (ANOVA) was adopted to examine the main effect of group (sprinters, long‐distance runners, untrained participants) on the total and relative muscle volume of MG, LG, and SOL. If a main effect was confirmed, a post hoc test (Tukey) was conducted. Statistical analyses were performed using the SPSS (version 20; IBM SPSS Statistics, Tokyo, Japan), with the level of statistical significance set at *α* = .05. All data are shown as mean and standard deviation. The normality of data was examined by Shapiro–Wilk test, and found that all data were normally distributed (*p* = .063–0.862).

## 3 RESULTS

**FIGURE 1:**
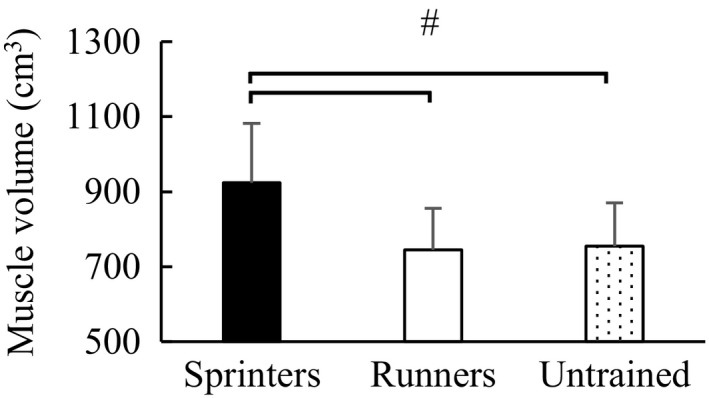
ANOVA analysis of total muscle volume among groups. ^#^Significant difference between groups (*p* < .05). Error bars are shown as *SD*. ANOVA, analysis of variance

One‐way ANOVA revealed a significant main effect of the total muscle volume among groups (*F* value = 25.564, *p* < .001). The subsequent post hoc test revealed that the total muscle volume was significantly larger in the sprinters (923.5 ± 158.8 cm^3^) than in the long‐distance runners (744.9 ± 110.8 cm^3^) (*p* < .001) (effect size = 1.35) and untrained participants (755.2 ± 115.8 cm^3^) (*p* < .001) (effect size = 1.25), while no significant difference was observed between long‐distance runners and untrained participants (*p* = .937) (Figure 1) (effect size = 0.09).

**FIGURE 2:**
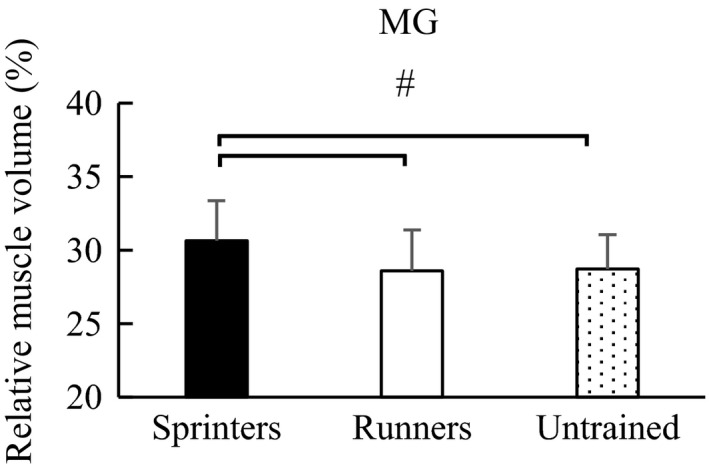
Post hoc analysis of relative muscle volume of MG among groups. ^#^Significant difference between groups (*p* < .05). Error bars are shown as *SD*. MG, medial gastrocnemius

**FIGURE 3:**
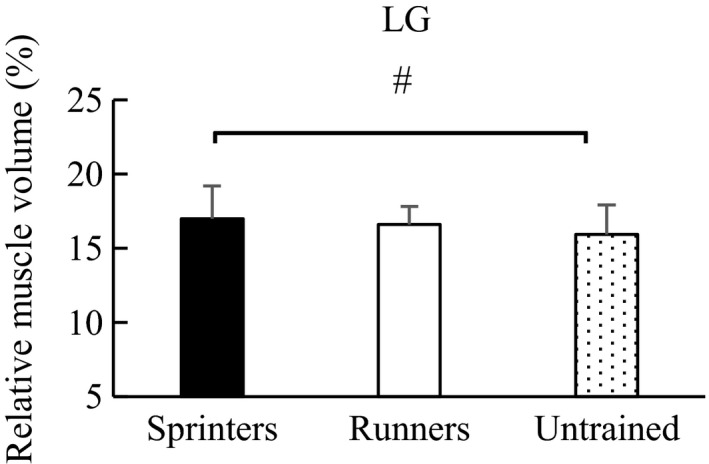
Post hoc analysis of the relative muscle volume of LG among groups. ^#^Significant difference between groups (*p* < .05). Error bars are shown as *SD*. LG, lateral gastrocnemius

**FIGURE 4:**
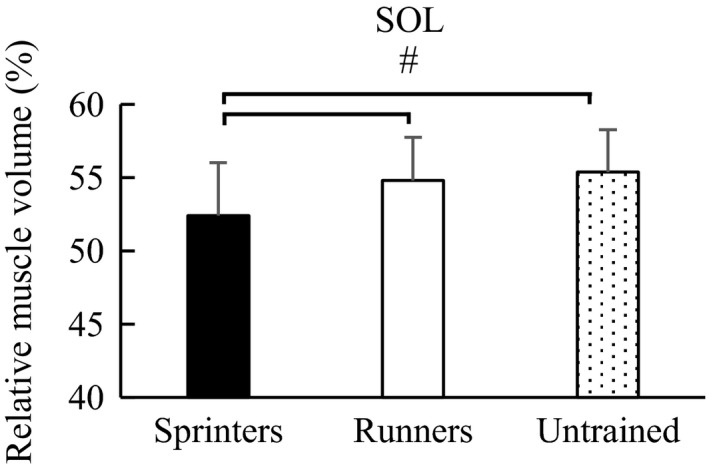
Post hoc analysis of the relative muscle volume of SOL among groups. ^#^Significant difference between groups (*p* < .05). Error bars are shown as *SD*. SOL, soleus

Similarly, regarding the relative muscle volume, one‐way ANOVA showed a significant main effect among groups: MG (*F* value = 8.498, *p* < .001), LG (*F* value = 3.367, *p* = .038), and SOL (*F* value = 10.702, *p* < .001). Post hoc test revealed that the relative muscle volume of MG was significantly larger in the sprinters (30.6 ± 2.7%) than in the long‐distance runners (28.6 ± 2.8%) (*p* = .001) (effect size = 0.73) and untrained participants (28.7 ± 2.3%) (*p* = .003) (effect size = 0.75). No significant difference was observed between the long‐distance runners and untrained participants (*p* = .972) (effect size = 0.04) (Figure 2). For LG, the relative muscle volume was significantly larger in the sprinters (17.0 ± 2.2%) than in the untrained participants (15.9 ± 2.0%) (*p* = .030) (effect size = 0.52). No significant difference was observed between the sprinters and long‐distance runners (16.6 ± 1.2%) (*p* = .646) (effect size = 0.22) and between the long‐distance runners and untrained participants (*p* = .238) (effect size = 0.43) (Figure 3). With respect to SOL, the relative muscle volume was significantly smaller in the sprinters (52.4 ± 3.7%) than in the long‐distance runners (54.8 ± 3.0%) (*p* = .002) (effect size = 0.43) and untrained participants (55.4 ± 2.9%) (*p* < .001) (effect size = 0.71), while no significant difference was observed between the long‐distance runners and untrained participants (*p* = .724) (effect size = 0.20) (Figure 4).

## 4 DISCUSSION

The purpose of this study was to examine whether the relative muscle volume of MG, LG, and SOL varies among participants through the comparison among participants that have different training status, that is, sprinters, long‐distance runners, and untrained participants. We showed that the relative muscle volumes of MG and LG were larger and those of SOL were smaller in the sprinters than in the other groups. These results indicate that the relative muscle volume can be different among participants.

A possible reason for the different relative muscle volume would be different muscle fiber types present in the triceps surae. Human MG and LG are composed of 50% fast‐twitch muscles and 50% slow‐twitch muscles, while human SOL is mainly composed of slow‐twitch muscles (Johnson et al., 1973). It is generally considered that muscle hypertrophy is prominent in fast than slow twitch fibers (Oishi et al., 2002; Shi et al., 2007). Taking these into account, it would be reasonable to expect that MG and LG are selectively hypertrophied with respect to SOL, and consequently, the relative muscle volume of triceps surae can differ. This hypothesis was partly supported by our results showing that the relative muscle volumes of MG and LG were larger and those of SOL were smaller in the sprinters than in the other groups.

A recent study examining the muscle volume of sprinters and long‐distance runners has reported contradicting results (Bex et al., 2017). Specifically, they reported that the relative muscle volume was similar between sprinters and long‐distance runners (Their Table 3B) while we found the significant difference. One of the possible reasons for this discrepancy is that the race of participants was different (Caucasian for Bex et al., (2017) and Japanese for the current study). In addition, because the extent of difference in muscle volume between sprinters and long‐distance runners reported in Bex et al., (2017) was much smaller (6%) than that reported in the current study (24%), there is a possibility that the participants recruited in the Bex et al., (2017) as the long‐distance runners had relatively hypertrophied triceps surae as well as sprinters or that the participants recruited as the sprinters had relatively small triceps surae. This would make it difficult to compare the muscle volume between sprinters and long‐distance runners. Finally, the number of subject in the previous study was only 19 while that in the current study was 126. Thus, the current data would reflect a more generalized tendency.

In the case of human experiments, it is difficult to measure muscle force produced by MG or SOL noninvasively because ankle plantar flexion torque, which can be measured in vivo, is generated by the sum of all plantar flexor muscles. To conduct in‐depth examinations such as tendon stiffness (Kubo, Kanehisa, & Fukunaga, 2001; Maganaris & Paul, 2002) and/or specific tension measurements (Erskine et al., 2011; Kawakami, Abe, Kuno, & Fukunaga, 1995), muscle force produced by each component such as MG or SOL should be obtained separately. The ratio of physiological CSA and/or muscle volume, which is an index of muscle force/joint torque, is needed to estimate the force produced by the individual muscle. Because MRI is required to obtain these indexes, the value experimentally observed in the previous study (Fukunaga et al., 1992) has been widely used in many studies (Burgess et al., 2009; Farris, Robertson, & Sawicki, 2013; Kubo et al., 2000; Lichtwark & Wilson, 2007, 2008; Takahashi, Gross, van Werkhoven, Piazza, & Sawicki, 2016). However, if the ratio of muscle size differs among participants, the aforementioned estimation may lead to overestimation or underestimation of calculated values. Based on the observed difference between groups (e.g., about 2% for MG), the magnitude of this underestimation/overestimation might be small. However, considering the individual difference (e.g., the minimum value was 22.5% while the maximum value was 35.4% for the MG in the sprinter group), this difference can affect substantially on the estimated muscle force. For example, if the plantar flexion torque is 100 Nm and moment arm is 5 mm, the calculated force is 2,000 N. If this force is divided by 22.5%, the calculated MG force is 450 N, whereas if this force is divided by 35.4%, the calculated MG force is 708 N. This difference can affect the calculated tendon stiffness or specific tension substantially.

Our study has several limitations. We first measured the muscle volume rather than physiological CSA, which is more closely related to muscle force (Fukunaga et al., 1992; Ikai & Fukunaga, 1968). Physiological CSA (i.e., transverse direction) are known to become larger after physical training (Erskine, Jones, Williams, Stewart, & Degens, 2010; McMahon, Morse, Winwood, Burden, & Onambélé, 2018; Morse, Thom, Mian, Birch, & Narici, 2007). Whether fascicle length (i.e., longitudinal direction) increases after physical training remains controversial; several studies reported increased fascicle length (Potier, Alexander, & Seynnes, 2009; Seynnes, de Boer, & Narici, 2007) while other studies reported no change (Ema, Wakahara, Miyamoto, Kanehisa, & Kawakami, 2013; Erskine et al., 2010; Morse et al., 2007; Scanlon et al., 2014; Seynnes et al., 2009). Taking these situations into account, it would be possible to assume that the observed larger muscle volume was mainly caused by the increased physiological CSA not by the increased fascicle length. Thus, the observed difference in muscle volume can reflect the difference in the physiological CSA among participants. Second, our research design was cross‐sectional study not longitudinal study and longitudinal study is basically considered as a more appropriate research design. This assumption is correct if the same number of participants are recruited with the same intervention period. However, because it is difficult (practically impossible) to conduct a long training intervention (several years) involving a large population (*N* > 100), a small number of participants (*N* = 10–20) with short training interventions (8–12 weeks) has been frequently applied. Considering the fact that the effect of physical training on muscle volume is gradual and requires a long duration to induce substantial muscle hypertrophy, the results of this cross‐sectional study recruiting many participants that have conducted years of specific physical activities can be useful.

We observed that the relative muscle volume was different among participants. Based on this finding, it is important to accurately measure the individual relative muscle size, especially for conducting in‐depth examinations such as muscle‐tendon interaction or specific tension. The observed different ratio of muscle size among groups might be explained by fiber type‐specific adaptation to physical exercises. Based on this speculation, there is a possibility that older participants may possibly have a different muscle size ratio compared to younger participants because the extent of muscle atrophy is also believed to be fiber type‐dependent (Nilwik et al., 2013; Snijders, Verdijk, & van Loon, 2009; Verdijk et al., 2007). This examination would be useful because we can extend the knowledge about whether the ratio of muscle size among synergists changes depending on training status and/or aging.

## 5 CONCLUSION

The relative muscle volumes of MG, LG, and SOL were different among sprinters, long‐distance runners, and untrained participants, indicating that the relative muscle volume is not necessarily constant among participants. Our results suggest that obtaining individualized information about muscle architecture is important to conduct an in‐depth examination, that is, muscle force distribution among synergists.

## CONFLICT OF INTEREST

No potential conflict of interest was reported by the authors.

## AUTHOR CONTRIBUTION

AF, TS, MT, AN, and TI designed this research project. AF, YT, YM, KT, HU, MO, TS, and MT collected and analyzed the data. All authors contributed to preparing the manuscript and approved the final version of this manuscript.

## ETHICAL STATEMENT

The purpose and risks of the study were explained to each participant, all of whom provided written informed consent. The Ethics Committee on Human Research of Ritsumeikan University approved the study (BKC‐IRB‐2016‐047). This study was conducted according to the code of ethics outlined in the Declaration of Helsinki.

## Data Availability

All available data are shown in the manuscript.
